# IQGAP1 Is a Novel CXCR2-Interacting Protein and Essential Component of the “Chemosynapse”

**DOI:** 10.1371/journal.pone.0023813

**Published:** 2011-08-18

**Authors:** Nicole F. Neel, Jiqing Sai, Amy-Joan L. Ham, Tammy Sobolik-Delmaire, Raymond L. Mernaugh, Ann Richmond

**Affiliations:** 1 Department of Veterans Affairs, Nashville, Tennessee, United States of America; 2 Department of Cancer Biology, Vanderbilt University School of Medicine, Nashville, Tennessee, United States of America; 3 Department of Biochemistry, Vanderbilt University School of Medicine, Nashville, Tennessee, United States of America; Agency for Science, Technology and Research (A*STAR), Singapore

## Abstract

**Background:**

Chemotaxis is essential for a number of physiological processes including leukocyte recruitment. Chemokines initiate intracellular signaling pathways necessary for chemotaxis through binding seven transmembrane G protein-couple receptors. Little is known about the proteins that interact with the intracellular domains of chemokine receptors to initiate cellular signaling upon ligand binding. CXCR2 is a major chemokine receptor expressed on several cell types, including endothelial cells and neutrophils. We hypothesize that multiple proteins interact with the intracellular domains of CXCR2 upon ligand stimulation and these interactions comprise a “chemosynapse”, and play important roles in transducing CXCR2 mediated signaling processes.

**Methodology/Principal Findings:**

In an effort to define the complex of proteins that assemble upon CXCR2 activation to relay signals from activated chemokine receptors, a proteomics approach was employed to identify proteins that co-associate with CXCR2 with or without ligand stimulation. The components of the CXCR2 “chemosynapse” are involved in processes ranging from intracellular trafficking to cytoskeletal modification. IQ motif containing GTPase activating protein 1 (IQGAP1) was among the novel proteins identified to interact directly with CXCR2. Herein, we demonstrate that CXCR2 co-localizes with IQGAP1 at the leading edge of polarized human neutrophils and CXCR2 expressing differentiated HL-60 cells. Moreover, amino acids 1-160 of IQGAP1 directly interact with the carboxyl-terminal domain of CXCR2 and stimulation with CXCL8 enhances IQGAP1 association with Cdc42.

**Conclusions:**

Our studies indicate that IQGAP1 is a novel essential component of the CXCR2 “chemosynapse”.

## Introduction

Chemokine receptors activate many intracellular signaling pathways through their coupling to G proteins. However, recent evidence suggests the importance of G protein-independent signaling pathways in the chemotactic response. The specific adaptor molecules that link activated chemokine receptors to these alternative signaling pathways are largely unknown. One might hypothesize that there is a dynamic exchange of proteins associated with the cytoplasmic domain of activated chemokine receptors that comprise a “chemosynapse” that serves a hub for signaling required for the chemotactic response. These interacting proteins effectively initiate organization of the actin cytoskeleton, receptor internalization and subsequent trafficking, and initiation of signaling needed for chemotaxis and other chemokine mediated cellular responses. The importance of chemokine receptors in a number of pathological conditions such as inflammation, angiogenesis, and cancer make them ideal for therapeutic targeting. Proteomic screening for the identification of novel interacting proteins is an ideal technique because it allows components of large signaling complexes within the cell to be elucidated. The technology not only allows identification of the proteins but the conditions under which these proteins interact under physiological conditions.

In the current study, we describe a novel proteomics technique which led to the identification of a novel CXCR2/IQGAP1 interaction. This approach has a number of benefits. First, proteins that bind CXCR2 indirectly can be identified using this method. Second, conformation-specific and modification-specific interactions can be identified. Third, interactions mediated through other intracellular domains of the receptor in addition to the carboxyl-terminus can be identified. Finally, immunoprecipitation of CXCR2-associating proteins in cells stimulated with ligand allows identification of dynamic and transient interactions. Use of this approach led to the identification of several novel CXCR2-interacting proteins that may be involved in the intracellular trafficking of the receptor, initiation of the chemotactic response, and activation of signaling pathways.

IQGAP1 was identified as a novel CXCR2-interacting protein. We propose that this interaction is an essential component of the dynamic “chemosynapse”. IQGAP1 is a major scaffolding protein involved in cytoskeletal organization and signaling through regulation of a number of cellular functions including adhesion, migration, and integration of complex signaling pathways within the cell. IQGAP1 is a 188 kDa protein that contains multiple domains (calponin-like actin binding domain, a WW domain that binds proline-rich motifs of multiple proteins, an IQ calmodulin-binding region, and a RasGAP domain) and localizes to the leading edge in migrating cells where it cross-links actin filaments [1], [2]. Interestingly, IQGAP1 contains a RasGAP homology domain but does not stimulate the GTPase activity. In fact, it inhibits the intrinsic GTPase activity of Rac1 and Cdc42, stabilizing them in their active forms [3], [4]. As expected, IQGAP1 has a fundamental role in cell motility. Expression of dominant negative IQGAP1, a form that is unable to bind Rac1 and Cdc42, or siRNA directed against IQGAP1 severely impairs cell motilifty and invasion [5]. Specifically, it plays an essential role in polarization of a migrating cell through its interactions with Rac1/Cdc42, APC, CLIP-170, actin and calmodulin (reviewed in [6]). Not only does IQGAP1 play a fundamental role in cell migration, it also serves as a scaffold for the MAP kinase signaling cascade [7], [8], suggesting that it may also play an important role in cell proliferation.

## Materials and Methods

### Cell culture

We have used HEK-293 and HL-60 cells in the experiments described herein. For all described experiments HL-60 cells were differentiated along the neutrophilic lineage as described previously [9]. Briefly, HL-60 cells were cultured in RPMI 1640 medium supplemented with 25 mM HEPES (pH 7.4), 10% fetal bovine serum (Atlanta Biologicals, Atlanta, GA), L-glutamine, 100 units/ml pen/strep (Mediatech, Inc., Herndon, VA). Cells were subcultured every 3–4 days to a cell density of 1×10^6^ cells/ml. To differentiate HL60 cells along the granulocytic lineage, cells were inoculated at a density of 2×10^5^ cells/ml in antibiotic-free medium containing 1.3% Dimethyl sulfoxide (DMSO) (endotoxin-free, Sigma) and cultured for 6–7 days. HEK293 cells were maintained in Dulbecco's Modified Eagle's Media with 10% fetal bovine serum and changed to serum free medium prior to assay.

### Purification of human neutrophils

Human blood from healthy donors was mixed with equal volumes of 3% dextran solution and set for sedimentation for 20 min at room temperature. The primary neutrophils were purified in a Ficoll-Hypaque gradient by centrifugation at 400× g for 40 min at room temperature.

### Immunoprecipitation of CXCR2 protein complexes

Normal rabbit IgG antibodies (Jackson Immunoresearch, West Grove, PA) and rabbit polyclonal anti-CXCR2 antibodies were coupled to NHS-activated Sepharose 4 Fast Flow matrix (GE Healthcare, Piscataway, NJ) at a 4∶1 ratio (mg antibody: ml matrix). Anti-CXCR2 affinity purified polyclonal antibody was generated in our laboratory and described previously [10]. Coupled antibody beads were blocked with ethanolamine (Sigma, St. Louis, MO) to minimize non-specific binding of proteins to matrix. For immunoprecipitations, HL-60 cells stably expressing CXCR2 were differentiated for 7 days in 1.3% DMSO and stimulated with vehicle (0.1% BSA/PBS) or 100 ng/ml CXCL8 for 1 min. Cells were resuspended in lysis buffer (50 mM Tris-HCl, pH 7.5, 0.05% Triton X-100, 300 mM NaCl), cleared by centrifugation, and pre-cleared with normal rabbit IgG-coupled beads. Precleared lysates were then incubated with normal rabbit IgG-coupled beads (Mock) or anti-CXCR2 rabbit antibody-coupled beads and associating proteins were eluted with 2X Laemmli sample buffer, heated for 65°C for 10 mins, loaded directly onto a 10% polyacrylamide gel with no stacker gel. The electrophoresis was continued until the dye front ran approximately 1 cm into the gel and proteins were stained with colloidal blue stain (Invitrogen, Carlsbad, CA). Excised gel bands were subjected to in-gel trypsin digest [11] and tryptic peptides submitted for LC/MS/MS analysis. Reverse co-immunoprecitation assays were performed in the same way with modifications as follows: Differentiated HL60 cells were cross-linked with 2 mM DSP (Cat# 22585, Pierce Biotechnology, Rockford, IL) immediately after stimulation with 100 ng/ml CXCL8. Then cells were lysed in 1x RIPA buffer supplemented with proteinase inhibitor cocktail (Sigma, St. Louis, MO) and phosphatase inhibitor cocktail 2 and 3 (Sigma, St. Louis, MO). Cell lysates were pre-cleared with normal rabbit IgG and protein A/G agarose beads (Santa Cruz Biotech, CA) and then precipitated with anti-IQGAP1 polyclonal rabbit antibody (Santa Cruz Biotech, CA) and protein A/G agarose beads.

### LC/MS/MS analysis and protein identification

One dimensional LC/MS/MS analysis was performed as described previously [12]. Briefly, analysis was performed using a Thermo Finnigan LTQ ion trap mass spectrometer and peptides were separated on a packed capillary tip (100 µm ×11 cm) with C18 resin (Monitor C18, 5 µm, 100 Å, Column Engineering, ON, Canada). MS/MS spectra of peptides was performed using data-dependent scanning in which one full MS spectrum, using a full mass range of 400-200 amu, was followed by 3 MS-MS spectra. Protein matches were searched with the Sequest algorithm (TurboSEQUEST v.27 (rev. 12) on a high speed, multiprocessor Linux cluster in the Vanderbilt Advanced Computing Center for Research) and preliminarily filtered using the criteria described previously [12]. Once the peptides were filtered based on these criteria, all matches that had less than two peptide matches were eliminated. These filtering criteria routinely achieved a false discovery rate of <1% in similar datasets. Protein matches were also validated by filtering the data through Protein Prophet in the Trans Proteomic Pipeline (Version: 4.0) (TPP v2.7 MIST rev.2, Build 200601131056).

### Immunofluorescence staining and confocal microscopy

Cells in serum-free RPMI were seeded on glass coverslips coated with 100 µg/ml human fibronectin (BD Biosciences, San Diego, CA) and stimulated globally with vehicle (0.1% BSA/PBS) or 100 ng/ml CXCL8 diluted in 0.1% BSA/PBS at 37°C for indicated times. Cells were fixed in 4% paraformaldehyde for 10 min, permeabilized in 0.2% Triton X-100/PBS for 5 min, blocked in 10% normal donkey serum for 30 min (Jackson Immunoresearch Laboratories, Inc., West Grove, PA). Anti-CXCR2 rabbit polyclonal (Mueller et al, 1994) and anti-IQGAP1 mouse monoclonal (Invitrogen, Carlsbad, CA) primary antibodies were added and incubated for 2 h at room temperature. After washing three times with 0.1% Tween 20/PBS, the coverslips were incubated with fluorescence-conjugated secondary antibodies for 1 h. After final three washes with 0.1% Tween 20/PBS, coverslips were mounted with ProLong Gold antifade reagent (Invitrogen, Carlsbad, CA). Confocal images were acquired using a LSM-510 Meta laser scanning microscope (Carl Zeiss, Thornwood, NY) with a 40X 1.3 numerical aperture oil immersion lens and images were processed by Photoshop software (Adobe Systems). Colocalization of CXCR2 with IQGAP1 was quantified using Metamorph Imaging System software package (Molecular Devices Corporation, Sunnyvale, CA). Threshold levels for all images were kept consistent among all images. Images were taken from six fields of view per time point in two separate experiments. The percent co-localization is indicative of the area of CXCR2 and stained fluorescent pixels overlapping that of IQGAP1.

### Construction of GST-IQGAP1 plasmids and preparation of recombinant GST-IQGAP1 protein from *Escherichia coli*

The pGEX-2T-IQGAP1-amino-terminus (NT) (aa 1-863) and –carboxyl-terminus (CT) (aa 864-1657) was a generous gift from Dr. David Sacks and were described previously [3]. To prepare the cDNA for GST-IQGAP1 1-160 and GST-IQGAP1 1-265 PCR was performed on pGEX-2T-IQGAP1-NT using the forward primers containing a 5′ BamHI site and a 3′ XhoI site. The same forward primer was used to prepare both fragments. The following primers were used for 1-160 amplification: forward-5′ ctctagggatccatgtccgccgcagacgaggtt 3′ and reverse- 5′ ctagctctcgagttagaacaggtacaaactgac 3′. The following reverse primer was used for 1-265 amplification: 5′ctagctctcgagttaagcctggtaaagtatat cctgg 3′. Fragments were amplified and digested with BamHI and XhoI, purified, and ligated into the pGEX-6P1 vector. All plasmids were purified using Sigma DNA maxiprep kits (Sigma, St. Louis, MO) according to the manufacturer's instructions. GST-fusion proteins were prepared from *Escherichia coli* as described previously. Briefly, cultures were inoculated and grown until OD600  = 0.6-0.8 and expression was induced with 10 µM isopropyl β-D-1-thiogalactopyranoside (IPTG) (Sigma, St. Louis, MO) 4 hs at 30°C. Bacteria were harvested, proteins extracted by sonication, and isolated by incubation with glutathione-agarose (Sigma, St. Louis, MO).

### Direct binding of purified IQGAP1-NT and GST-CXCR2 carboxyl-terminus

The GST tag was cleaved from purified GST-IQGAP1-NT coupled to glutathione-agarose by incubating with thrombin (10 units/1 mg total protein) (GE Healthcare, Piscataway, NJ) for 16 h at 25°C. Cleaved protein was incubated with Benzamidine Sepharose 4 Fast Flow Matrix (GE Healthcare, Piscataway, NJ) to remove thrombin. Constructs for glutathione *S*-transferase (GST) fusion proteins of the C-terminal residues of CXCR2 were generated previously [13]. Glutathione agarose beads coupled to GST or GST-CXCR2 carboxyl-terminus and reaction tubes were blocked with 1% BSA for 1 h at 25°C prior to binding assay. 50 µg total GST-fusion proteins on beads were incubated with 10 µg purified IQGAP1-NT for 1 h at 4°C in binding buffer (50 mM Tris-HCl, pH 7.5, 300 mM NaCl, 0.01% Triton X-100). Beads were washed four times with binding buffer. Bound proteins were eluted with 2X Laemmli sample buffer and subjected to SDS-PAGE and western blot analysis using an anti-IQGAP1 rabbit polyclonal antibody directed against the amino-terminus of the protein (Santa Cruz Biotechnology, Inc., Santa Cruz, CA).

### Chemotaxis Assay (Boyden chamber)

Chemotaxis assays were performed as previously described [13]. Briefly, HEK293 cells were trypsinized, washed and resuspended in serum containing medium. Cells were maintained at 37°C, 5% CO_2_ with rotation for 2 h and then cells were washed with 0.1% BSA/serum-free medium and re-suspended in the same medium. A 10-µm pore of polybrene filter was pre-coated with 2 mg/ml collagen IV (Sigma) and the Boyden chamber was assembled as described in manufacturer's protocol. The lower wells were filled with different concentrations of CXCL8 (0–25 nM) or EGF (0–50 nM) and upper wells were loaded with 10^5^ cells in 200 µl. Cells were allowed to migrate in the assembled chamber for 4-5 h at 37°C, 5% CO_2_. Cells on the filter were fixed and stained with crystal violet, viewed and counted under a 20x microscope objective. Assays were repeated a minimum of 3 times with duplicate wells at each concentration and 5 fields were counted per well

### Induction of Cell Polarization in a Zigmond Chamber

Purified cells (human primary neutrophils or differentiated HL60 cells) were seeded on a coverslip pre-coated with 10 mg/ml human fibronectin. The Zigmond chamber was assembled according to the manufacture's suggestions. The CXCL8 gradient was generated between the serum-free RPMI1640 medium and medium containing CXCL8 (25 ng/ml). Cells on the coverslips were induced to polarize under the CXCL8 gradient for 20 min at 37°C and then immediately fixed in 4% paraformaldehyde for 10 min at room temperature. Cells were washed three times with PBS and stored in this buffer at 4°C until use.

## Results

### Development of a proteomics approach to identify novel CXCR2-interacting proteins

In order to identify proteins that differentially associate with the un-stimulated receptor versus the activated receptor it was important to immunoprecipitate receptor-protein complexes from a physiologically relevant cell type. The analysis was conducted in the HL-60 cell line differentiated into the human neutrophil lineage because CXCR2 is essential for the inflammatory response due to its involvement in neutrophil recruitment. Differentiated HL-60 cells naturally express low levels of CXCR2. In order to maximize co-immunoprecipitating proteins, however, CXCR2 was stably over-expressed in the cell lines used in these experiments. An additional obstacle of performing these analyses is the presence of high amounts of IgG from immunoprecipitation which makes it difficult to detect spectra of peptides from less abundant proteins. In order to address this problem, normal rabbit IgG and anti-CXCR2 rabbit antibodies were covalently coupled to Sepharose beads. This allowed protein complexes to be eluted from the beads without IgG contamination.

**Figure 1:**
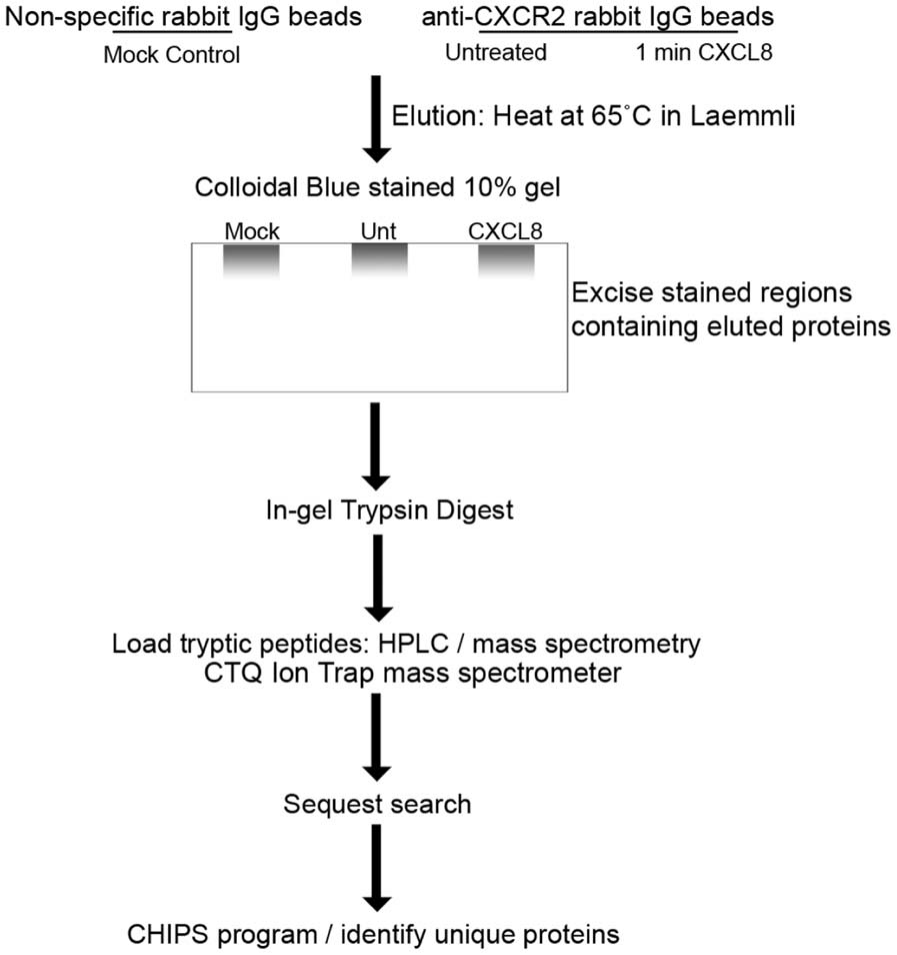
Schematic of representation of proteomics approach used to identify novel CXCR2-interacting proteins.

Cells were stimulated with either vehicle or 100 ng/ml CXCL8 for 1 min and lysed in a mild buffer in order to maintain weak interactions within protein complexes. Normal rabbit IgG- (mock control) and anti-CXCR2-coupled beads were then used to immunoprecipitate complexes from lysates and eluted using Laemmli sample buffer. Eluted proteins were then loaded directly onto a polyacrylamide resolving gel and allowed to run into the gel approximately 1 cm. Protein bands were stained with colloidal blue stain and excised from the gel. Tryptic peptides were generated by in-gel trypsin digest and subjected to LC/MS/MS analysis. Proteins were identified using the cluster version of the SEQUEST algorithm [14] using the human subset of the Uniref100 database (www.uniprot.org). Detailed methodology is described in the Materials and Methods section above. Unique proteins in each group were identified using an in-house Vanderbilt database program called CHIPS (Complete Hierarchical Integration of Protein Searches). This allowed non-specific identifications from the mock control immunoprecipitations to be subtracted from the two experimental groups. A schematic of the approach used to identify novel CXCR2-interacting proteins is shown in Figure 1. Some of the consistently identified CXCR2-associating proteins identified by this method with the corresponding number of peptides and frequency are listed in Table 1 and the interacting proteins identified from untreated vs. CXCL8-stimulated cells are listed in Table 2.

**Table 1:** Associating CXCR2 proteins identified with proteomics.

Protein identified	Total number of peptides	Number of runs identified
IQGAP1	6	3/4
14-3-3 gamma	4	2/4
VASP	5	3/4
P21 Arc	3	2/4
LASP-1	4	3/4
kinesin	4	3/4
Dynein heavy chain 5	6	4/4
Valosin-containing protein(VCP)	15	4/4
Nipsnap	2	2/4

**Table 2:** List of identified proteins from untreated and CXCL8 stimulated cells in LC/MS/MS analysis.

	Untreated	CXCL8 treated	Both Untreated and CXCL8 treated
**Actin cytoskeleton**	P21-ArcGelsolinPlastin	Vasodilator-stimulated phosphoprotein (VASP)	Talin 1Arp 2/3 subunit 2Lasp-1
**Intracellular trafficking**	Rab7Annexin 1Kinesin light chain-2Valosin-containing protein (VCP)	Secretory carrier membraneprotein 2 (SCAMP2)Nipsnap homolog 1Dynein heavy chain 5	Clathrin heavy chain 1
**Signaling scaffolding**	14-3-3 gamma	Similar to LIN-41	YWHAZ (14-3-3 zeta)IQ motif-containing GTPase activating protein 1 (IQGAP)
**Other**	Hsp 90Hsp 75Chaperonin TCP1	PSMA2 (proteosome subunit)26S proteosome subunit 9	

### IQGAP1 is a novel CXCR2 interacting protein

**Figure 2:**
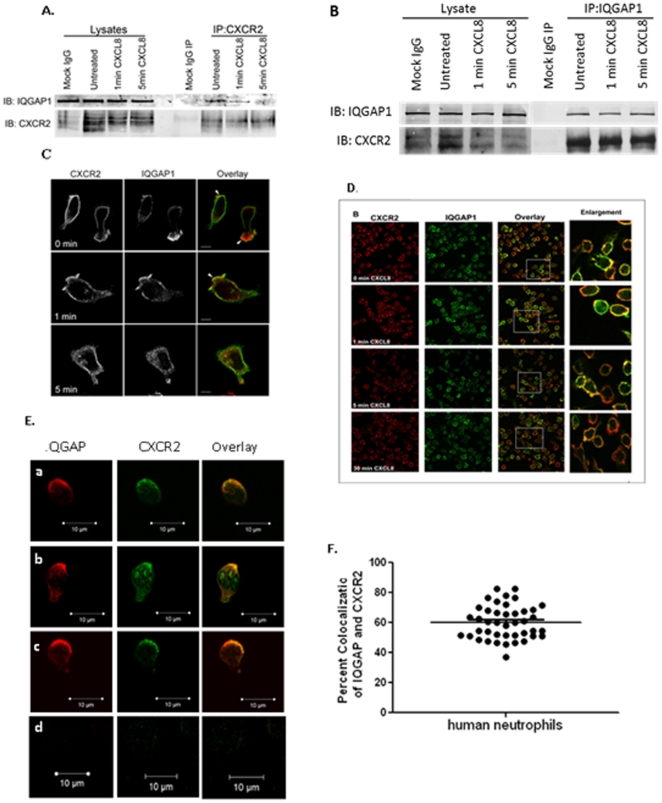
IQGAP1 is a novel CXCR2 interacting protein. (A) IQGAP1 co-immunoprecipitates with CXCR2. Lysates from differentiated HL-60 cells expressing CXCR2 stimulated with vehicle (Mock, Untreated) or cells stimulated with 100 ng/ml CXCL8 for 1 min or 5 min were incubated with either normal rabbit IgG- (Mock IgG) or rabbit anti-CXCR2 antibody-coupled sepharose. Beads were washed and immunoprecipitated proteins were eluted with Laemmli sample buffer. Samples were analyzed by SDS-PAGE and western blot (IB) for CXCR2 and IQGAP1. (B) CXCR2 co-immunoprecipitates with IQGAP1-reverse co-immunoprecipitation. Cell lysates were prepared as described above and incubated with either normal rabbit IgG (Mock IgG) or polyclonal rabbit anti-IQGAP1 antibody. Immunoprecipitated proteins were analyzed by SDS-PAGE and western blot for IQGAP1 and CXCR2. (C & D) Co-localization of CXCR2 and IQGAP1. Immunofluorescence confocal images of CXCR2 and IQGAP1 staining in differentiated HL60 cells stably expressing CXCR2 and stimulated with vehicle (0 min) or 100 ng/ml of CXCL8 for 1 min, 5 min, and 30 min. Cells were stained with rabbit polyclonal anti-CXCR2 and mouse monoclonal anti-IQGAP1 antibodies, and incubated with species specific Cy2- and Cy3-conjugated secondary antibodies. Co-localization is seen at each time point. Overlay images are pseudo-colored where green is IQGAP1 and red is CXCR2. Image represents a single Z-section of 0.28 µm. Insets are enlarged 2X from original images. (E) Co-localization of IQGAP1 and CXCR2 in human neutrophils. Purified human neutrophils were induced to polarize in a Zigmond chamber under a gradient of CXCL8 (the arrows indicate the direction of gradient) for 20 min at 37°C. Panels a-c show three representative confocal immunofluorescence images of cells where cells are stained with antibodies against IQGAP1 (red) and CXCR2 (green). Panel d shows images of cells stained with normal IgG (isotype control). Scale bar  = 10 µm. Quantitation of percent co-localization of IQGAP1 and CXCR2 was plotted in panel F.

The signaling scaffolding protein IQGAP1 was consistently identified with both the un-stimulated and activated receptor immunoprecipitations using this approach. A total of six peptide spectra were identified for IQGAP1 and spectra were identified in three out of the four replicate experiments (Table 1). The interaction of IQGAP1 with CXCR2 was verified by immunoprecipation followed by western blot analysis (Figure 2A and 2B). In support of the proteomics analyses, western blot analysis shows an apparently equal amount of immunoreactive IQGAP1 co-IPs with CXCR2 in untreated cells and cells stimulated with CXCL8 for 1 min. Interestingly, IQGAP1 no longer co-IPs with CXCR2 following 5 mins of CXCL8 stimulation (Figure 2A). A reverse co-immunnoprecipitation with anti-IQGAP1 antibody followed by Western blot for CXCR2 was performed. This assay confirmed the association of IQGAP1 and CXCR2 (Figure 2B). Intracellular localization of IQGAP1 was also examined upon CXCL8 stimulation by immunofluorescence staining and confocal microscopy. In unstimulated cells, IQGAP1 is localized just below the plasma membrane and accumulates in membrane ruffles with CXCR2 upon 1 min of CXCL8 stimulation (Figure 2C). Consistent with the immunoprecipitation experiments, confocal analyses demonstrate that upon global ligand stimulation with IL-8, IQGAP1 localizes to membrane where CXCR2 is also localized over a time course of 30 minutes (Figure 2C and 2D). In confocal microscopy experiments analyzing the co-localization of IQGAP1 and CXCR2 in polarized normal human neutrophils responding to a gradient of IL-8 chemokine in Zigmond chamber assays, immunofluorescence staining indicated that about 60% of the IQGAP1 was observed to be overlapping with CXCR2 in confocal microscopy. Most of this overlap was concentrated on the leading edge of the cells polarized toward the direction of a CXCL8 gradient (Figure 2E and 2F). Of note, while there is a dimunition of co-immunoprecipitating IQGAP1 and CXCR2 by 5 minutes after ligand stimulation, in confocal microscopy experiments, these two proteins continue to co-localize at the membrane, suggesting that though they may no longer physically associate, they remain in the same proximity at the membrane at the 5 minute time point.

### CXCR2 interacts with the amino-terminus of IQGAP1 specifically through amino acids 1-160

**Figure 3:**
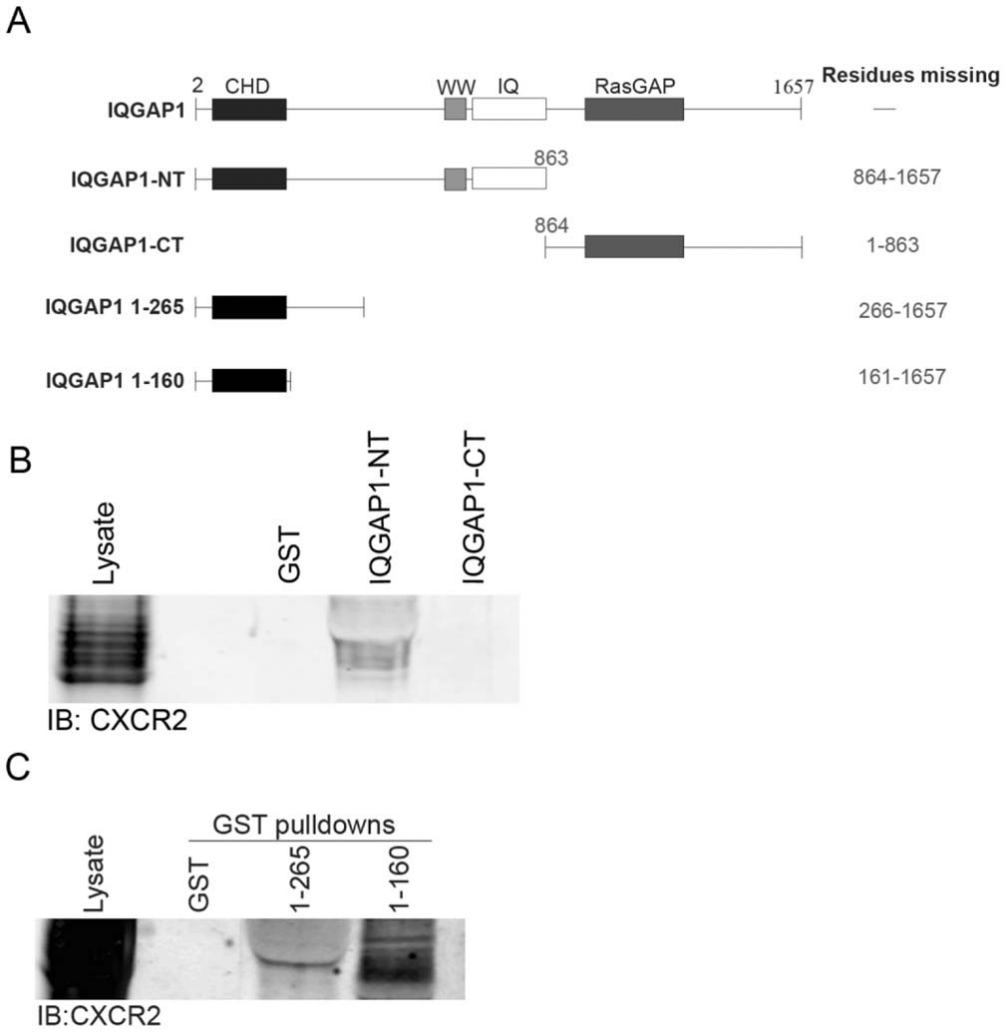
CXCR2 interacts with the amino-terminus of IQGAP1 specifically through amino acids 1–160. (A) Domains contained in the GST-IQGAP1 fusion construct. Western blot analysis with anti-CXCR2 antibody of differentiated HL-60 CXCR2 lysates (Input) and eluates from (B) GST, GST-IQGAP1-N-terminus (NT), and IQGAP1-C-terminus (CT) pull-down reactions and (C) GST, GST-IQGAP1-1-265, and -1-160. Data shown are representative of three separate experiments.

To determine the domain of IQGAP1 that interacts with CXCR2, GST-IQGAP1-amino-terminus (NT) (residues 1–863) and –carboxyl-terminus (CT) (residues 864–1657) fusion proteins were produced (Figure 3A). Pull-down reactions were performed using these proteins and lysates from differentiated HL-60 cells stably expressing CXCR2. As shown in Figure 3B, the amino terminus of IQGAP1 interacts with CXCR2 from HL-60 cells. GST fusion proteins of successively smaller domains within the amino-terminus of IQGAP1 were generated to further define the interaction domain. Amino acids 1–265 and 1–160 were both able to efficiently interact with CXCR2 from HL-60 cells (Figure 3C). GST fusion proteins containing residues 1-44 and 160–431 were also generated and used in pull-down reactions. These two fusion proteins failed to interact with CXCR2 (Supplemental Data Figure S4). These data suggest that a region of IQGAP1 located between residues 44 and 160 is likely the CXCR2-interaction domain.

### CXCR2 directly interacts with the amino-terminus of IQGAP1

**Figure 4:**
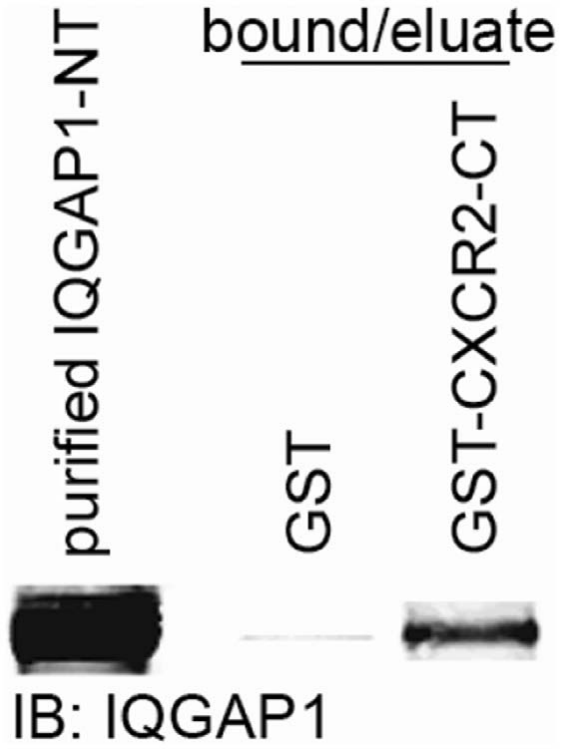
Purified IQGAP1/N-terminus binds directly to GST CXCR2/C-terminus. Western blot analysis of purified IQGAP1/N-terminus (NT) and purified protein bound to glutathione Sepharose beads with GST alone or GST-CXCR2/C-terminus (CT). Briefly, fusion protein-bound glutathione beads were blocked with 1% BSA for 1 h at room temp and incubated for 1 h at 4°C with 10 µg purified IQGAP1/N-terminus in binding buffer. Beads were washed 3X with binding buffer and bound protein eluted and analyzed by SDS-PAGE and western blot (IB) for IQGAP1. Data shown are representative of three separate experiments.

We next sought to determine whether the IQGAP1 interaction with CXCR2 is direct or occurs through an intermediate adaptor protein. To investigate this, recombinant IQGAP1-amino-terminus (NT) and GST-CXCR2-carboxyl-terminus were purified. These proteins were then used in direct binding assays in order to assess whether the two proteins can interact in the absence of other cellular proteins. There is only a slight association of IQGAP1 with GST alone, which is not unexpected based upon the potential for some non-specific binding to GST alone. These assays demonstrated that purified IQGAP1-NT and GST-CXCR2 carboxyl-terminus are able to bind *in vitro* (Figure 4). These data suggest that CXCR2 directly interacts with the amino-terminus of IQGAP1.

### Interaction of IQGAP1 with Cdc42 is enhanced by CXCL8 stimulation

**Figure 5:**
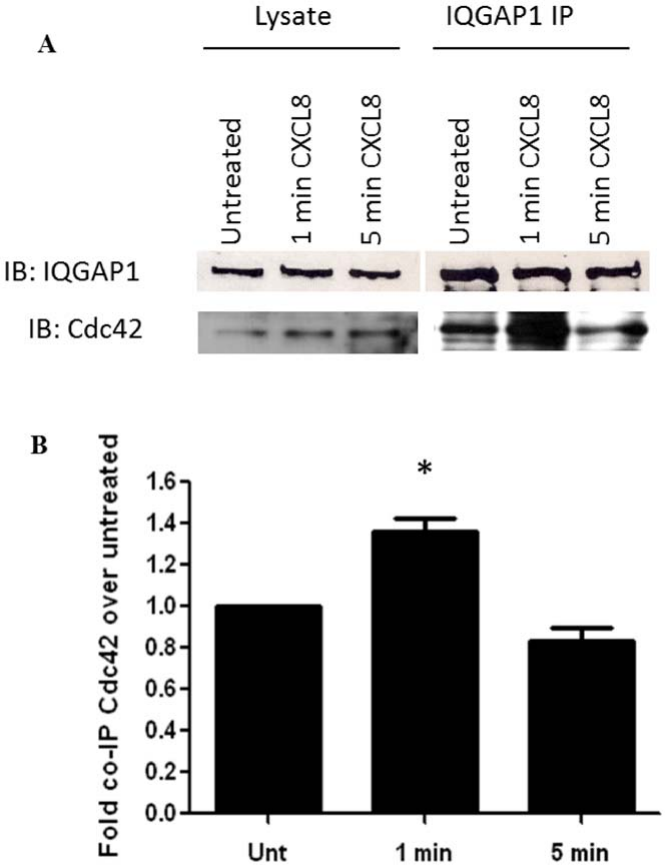
Interaction of IQGAP1 with Cdc42 is enhanced by CXCL8 stimulation. Lysates from cells stimulated with vehicle (Mock, Untreated) or cells stimulated with 100 ng/ml CXCL8 for 1 minute or 5 minute were incubated with either normal rabbit IgG- (Mock IgG) or rabbit anti-IQGAP1 antibody and Protein A/G agarose beads. Immunoprecipitated proteins were eluted with Laemmli sample buffer (A) Representative western blot of samples analyzed by SDS-PAGE and immunoblotted (IB) for IQGAP1 and Cdc42 (B) Quantitation using densitometry of fold increase in amount of Cdc42 that co-immunoprecipitated with IQGAP1 following CXCL8 stimulation over amount of Cdc42 that co-immunoprecipitates from lysates of untreated cells. Graph represents mean fold increase ± s.e.m. from three separate experiments. There is a statistically significant increase in the amount of Cdc42 that co-immunoprecipitates with IQGAP1 following 1 minute of CXCL8 stimulation as represented by the asterisk (p-value <0.05, Mann Whitney U-test).

A number of studies have demonstrated that the interaction of IQGAP1 with the small GTPases Rac and Cdc42 is affected by various different stimuli such as cell-cell adhesion [4], [15], [16], cell-matrix adhesion [16], and Ca^2+^ signaling [17]. Cdc42 binds IQGAP1 only in its GTP bound state [18]. Because Cdc42 is activated upon CXCR2 stimulation, we sought to investigate whether the association of Cdc42 with IQGAP1 was altered upon CXCL8 stimulation. We examined if the association of IQGAP1 with Cdc42 was altered upon CXCL8 stimulation. To investigate this, we immunoprecipitated IQGAP1 from differentiated HL-60 cells expressing CXCR2 stimulated with CXCL8 and examined association of Cdc42 by western blot analysis. These experiments demonstrated that co-immunoprecipitation of Cdc42 with IQGAP1 is slightly enhanced with CXCL8 stimulation (Figure 5A). This experiment was repeated three times with similar results demonstrating an average of a 1.36±0.1 fold increase in co-immunoprecipitated Cdc42 following 1 min of CXCL8 stimulation as quantitiated by densitometry (Figure 5B). This establishes a potential functional link between CXCR2 activation and modulation of IQGAP1 activities within the cell.

### Expression of IQGAP1 1-160 (CXCR2-interacting domain) impairs CXCR2-mediated chemotaxis

**Figure 6:**
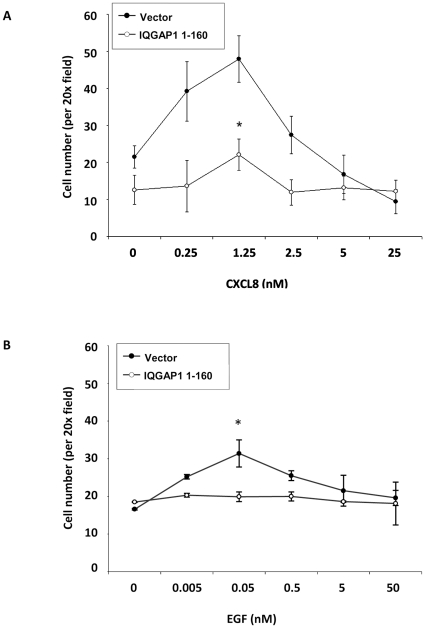
Boyden chamber assay assessing chemotaxis of HEK293-CXCR2 cells stably expressing empty vector or IQGAP1 1–160. HEK293-CXCR2 cells, with IQGAP1 1-160 and without (vector only) were stimulated with CXCL8 (A) or EGF (B) and assayed using a Boyden chamber to determine chemotaxis. A statistically significant difference between cells expressing vector and IQGAP1 1-160 was found and is represented by the asterisk (p-value < 0.001 (A) and  = 0.0111 (B); ANOVA). Graph represents the average cell number per 20X field ± s.e.m. and is representative of three separate experiments.

To determine whether the IQGAP 1-160 domain (CXCR2 interaction domain) will compete for CXCR2 and reduce the endogenous interaction between CXCR2 and IQGAP1 we transfected HEK293 cells stably expressing CXCR2 with an expression construct encoding IQGAP1 1-160. After verifying expression of the 1-160 domain, we compared CXCR2-mediated chemotaxis of the cells expressing or not expressing the 1-160 domain using a modified Boyden chamber assay. Expression of this fragment significantly inhibited CXCR2-mediated chemotaxis of HEK293 cells as compared to cells transfected with empty vector (Figure 6). To determine whether the inhibition was specific for the CXCR2 mediated chemotaxis, we also examined EGF mediated chemotaxis in the same cells. Expression of the IQGAP1 1-160 fragment in HEK-293 cells expressing CXCR2 also resulted in significant inhibition of EGF-mediated chemotaxis in a Boyden chamber chemotaxis assay, suggesting that the 1-160 fragment also affects the ability of other receptors to effectively use IQGAP1 to mediate chemotaxis.

After extensive experimentation, we are unable to offer proof that the 1-160 fragment inhibits chemotaxis by directly blocking the binding of full length IQGAP1 to CXCR2 or the EGFR. In co-immunoprecipitation experiments, overexpression of IQGAP1 1-160 fragment in HEK-293 cellls or inclusion of the 1-160 fragment in the dHL60 lysate will not block the co-immunoprecipitation of CXCR2 with full length IQGAP1 (Supplemental data Figures S1 and S2). We postulated that if the 1-160 fragment could bind to full length IQGAP1, then there might be a conformational change in IQGAP1 that would prevent its binding to Cdc42. However, we observed that the 1-160 fragment does not block the association of IQAGAP with Cdc42 (Supplemental Data Figure S3). In retrospect, this is likely because the region of self-association for IQGAP1 requires amino acids 763–863 and not the N terminal domain. From our data it appears that though the 1-160 fragment of IQGAP1 binds CXCR2, and expression of 1-160 interrupts chemotaxis, IQGAP1 will continue to co-immunoprecipitate with CXCR2. This is likely due to the ability of IQGAP1 to bind other adaptor proteins that associate with CXCR2. There are a number of plausible explanations for how the 1-160 fragment might function to block CXCL8 and EGF mediated chemotaxis which we address below in the discussion.

## Discussion

We have developed a highly effective approach to identify novel dynamic chemokine receptor-interacting proteins. This approach allows protein complexes to be isolated from cells following various periods of ligand stimulation and has the potential to temporally define the components of chemokine receptor-associating complexes within the cell. This has important implications for the current understanding of the chemotactic response. In addition to IQGAP1, the following proteins were found: 14-3-3 gamma, p21-Arc, a subunit of an Arp 2/3 protein complex, which also localizes to sites of actin polymerization [19], vasodilator stimulated phosphoprotein (VASP), LIM and SH3 domain protein 1 (LASP-1), microtubule motor proteins such as dynein, valosin-containing proteins, Rab39 and Nipsnap (Table 1). We have previously characterized the association of CXCR2 with VASP and LASP-1 20], both of which are important in cytoskeletal organization and cell motility. The Ena/VASP family of proteins can enhance actin polymerization by recruiting profilin-actin complexes to sites of acting remodeling, such as the lamellipodia in migrating cells [21], [22], while Lasp-1 is a component of focal adhesions that has recently been shown to play an important role in cell migration and cell survival [23], [24], [25]. Each of these actin regulating proteins may provide a link between the activated chemokine receptor and the acting cytoskeleton.

Two components of microtubule motor proteins were also among the proteins identified with this approach. These proteins may play a role in the intracellular trafficking of chemokine receptors, as well as in signaling by transporting signaling proteins to appropriate cellular locations. For example, kinesins are microtubule motor proteins that are involved in transporting vesicles and organelles along microtubules. Interestingly, it was found that 14-3-3 interacts with kinesin light chain-2 in a phosphorylation-dependent manner [26]. Dynein heavy chain, another microtubule motor protein, was also identified in complexes from stimulated cells. Furthermore, microtubules are known to play a role in the endocytosis of other G protein-coupled receptors such as beta2-adrenoceptor [27] and m3-muscarinic receptors [28]. Additional proteins that may play a role in the intracellular sorting of internalized CXCR2 were among the novel CXCR2-interacting proteins. Valosin-containing protein is a molecular chaperone that is involved in the ubiquitin-proteosome degradation pathway [29], [30]. Rab39 is a Golgi-associated GTPase that can facilitate endocytosis [31]. Nipsnaps are a family of proteins that have a potential role in vesicular trafficking because of their homology to synaptosomal-associated proteins (SNAP) [32]. In addition, NIPSNAP4 was shown to interact with the *Salmonella enterica* factor SpiC, which inhibits lysosomal maturation [33].

Among the proteins identified in the untreated samples was 14-3-3 gamma. 14-3-3 gamma is a signaling adaptor molecule that can bind regulators of G-protein signaling (RGS) proteins and inhibit their activity [34], [35]. RGS proteins are GTPase activating proteins (GAPs) that specifically activate the Gα subunit [36], [37]. It has been previously shown that RGS12 interacts with CXCR2 through its PDZ (PSD95/DlgA/ZO-1) domain [38]. Therefore, the interaction of CXCR2 with 14-3-3 gamma represents a potential novel link to signaling pathways through its function as an adaptor molecule that is a known regulator of RGS proteins.

IQGAP1 binds to CXCR2 in the absence of CXCL8 stimulation and after prolonged ligand stimulation this interaction is reduced. It is likely that once CXCR2 internalizes, IQGAP1 dissociates from the receptor. CXCL8 stimulation induces CXCR2 phosphorylation, Cdc42 activation and polarization, and a rise in intracellular Ca^2+^ levels [10] [39] [40]. Activated Cdc42 and Ca^2+^/calmodulin both modulate IQGAP1 activities and binding partners (reviewed in [1], [6]. It has been previously demonstrated that inhibition of Ca^2+/^calmodulin by the cell-permeable inhibitor CGS 9343B impairs the Ca^2+^-dependent interaction of E-cadherin with IQGAP1 [41]. Therefore, we are interested in whether CXCR2 phosphorylation, Cdc42 activity, and Ca^2+^/calmodulin influence CXCR2/IQGAP1 binding. In the future, it will be of interest to express dominant negative and activated Cdc42 mutants and determine whether the interaction between CXCR2 and IQGAP1 is enhanced or disrupted. Additionally, investigation into the effects of inhibition of CXCR2 phosphorylation using phosphorylation-deficient receptor mutants and inhibition of Ca^2+^/calmodulin through use of a cell-permeable calcium chelator on the CXCR2/IQGAP1 interaction will be of interest.

It is of interest that CXCR2 interacts with IQGAP1 between amino acids 44 and 160 because this region also contains the WW domain where calmodulin binds. This region of IQGAP1 is necessary and sufficient for its high affinity interaction with F-Actin [42]. Studies utilizing point mutations in the WW domain demonstrated that the IQGAP1 interaction with F-Actin is essential for its role in the promotion of cell motility [43]. In addition, interaction of IQGAP1 with calmodulin has been shown to inhibit the binding of IQGAP1 to actin [44]. Therefore, it is possible that CXCR2 competes with actin for binding to the WW domain of IQGAP1, similar to the phenomenon seen with calmodulin.

We describe a novel and effective technique to identify components of dynamic chemokine receptor protein complexes within the cell. A number of interesting proteins have been identified that potentially regulate chemokine receptor function. These proteins range in function from intracellular trafficking to cytoskeletal modification. One of the CXCR2 interacting proteins identified is IQGAP1. We have identified amino acids 1–160 of IQGAP1 as the CXCR2 interaction domain. This domain can be utilized to antagonize the CXCR2 mediated chemotaxis. However, after extensive experimentation, we have concluded that though the 1–160 fragment may inhibit CXCL8 and EGF mediated chemotaxis, we are still able to co-immunoprecipitate IQGAP1 with CXCR2 in the presence of the 1–160 fragment. Several scenarios may be considered for the mechanism of action of the 1–160 fragment, of which scenario 3 appears most promising.

1. CXCR2 might bind the 1–160 fragment and the 1–160 fragment would also bind a full length IQGAP1 monomer, and in this conformation the IQGAP1 would be unable to dissociate and bind actin when ligand stimulates the binding of activated Cdc42 to IQGAP1. In this scenario, the receptor continues to co-immunoprecipitate with full length IQGAP1, even though the full length IQGAP1 may no longer bind receptor. In this case when the 1–160 fragment binds IQGAP1, it works as a dominate negative for IQGAP1 function such that IQGAP1 is no longer able to become fully activated to dissociate and bind and bundle actin. However, Fukata et al have shown that a 1–216 GST fragment of IQGAP1 will not pull down endogenous IQGAP1 [45]. Also Ren et al demonstrated that the self-association region for IQGAP1 requires amino acids 763–863[46]. Thus it is doubtful that the 1–160 fragment is binding full length IQGAP1.
2. The inhibitory effect of the 1–160 fragment might result from binding of the WW domain in the 1–160 fragment to F-actin, thus blocking the binding of F-actin to full length IQGAP1. However, Mateer et al have shown that a 1–210 fragment of IQGAP1 binds F-actin in a mole to mole ratio and the 1–210 fragment of IQGAP1 co-localizes with the cortical actin, but not with the actin in stress fibers[42]. Thus, if present in sufficient levels, the 1–160 fragment of IQGAP1, by binding to F-actin, might block the formation of IQGAP1 dependent ligand stimulated stress fiber formation involved in cell motility. This scenario is less likely if the relative abundance of F-actin is much greater than the abundance of IQGAP1 or the 1–160 fragment.
3. The 1–160 fragment of IQGAP1 might bind CXCR2, displacing direct binding of full length IQGAP1 and blocking the downstream signaling needed for chemotaxis. However, in this scenario full length IQGAP1 would continue to co-immunoprecipitate with CXCR2 based upon the ability of IQGAP1 to bind other proteins that associate with adaptor proteins that bind CXCR2. However, in this scenario, the “tag along” binding would not allow functional activation of ligand mediated signaling through CXCR2/IQGAP1 interaction.
4. The 1–160 fragment might inhibit CXCR2 and EGFR mediated chemotaxis by binding to WASP are replacing the ability of IQGAP1 to bind WASP, thus preventing its ability to generate branched actin filaments needed for chemotaxis. WASP normally binds the WW domain of IQGAP1 through its EVH1 domain then WASP binds Arp2/3 through its verprolin-connecting-acidic domain and CDC42 through its gap related domain [47]. CDC42 also binds IQGAP1 in its gap related domain. Since the 1–160 fragment of IQGAP1 would directly bind to WASP [47], [48], this would prevent the binding of WASP to full length IQGAP1 and block receptor mediated signaling. Thus while CXCR2 might still bind full length IQGAP1, upon ligand induced dissociation from the receptor, the full length IQGAP1 would not be able to bind WASP to mediate Arp2/3/Cdc42 promoted branched actin filament polymerization, since the WASP would remain pre-bound to the 1–160 fragment. Of note, the 1–160 fragment did not reduce the association of IQGAP1 with Cdc42 through its GRD domain, but this doesn't rule out the possibility that there would be an impairment of branched actin filament polymerization in response to chemokine in the presence of the 1–160 fragment(Supplemental data Figure S3).

We postulate that under normal circumstances, when IQGAP1 is bound to CXCR2, IQGAP1 is unable to bind actin and when ligand stimulates CXCR2 mediated events, including activation of Cdc42, the IQGAP1 binds activated Cdc42, enabling IQGAP1 to undergo a conformational change such that it is released from the receptor so that it can dimerize and bind F- actin through the WW domains, cross-link actin filaments, and mediate chemotaxis. This concept is further supported by studies utilizing point mutations in the WW domain (within 44 and 160 of IQGAP1) result in a loss of IQGAP1 mediated cell motility [42]. Moreover, IQGAP1 binding of calmodulin places IQGAP1 in a conformation that not only inhibits the binding of IQGAP1 to actin but also inhibits its ability to bind to other partners needed for chemotaxis [42].

In agreement with our work shown here, Yamaoka-Tojo et al., showed that VEGF binding to VEGFR2 stimulates the direct binding of IQGAP1 to the cytoplasmic domain of the VEGFR2 (aa 790-958) and association of Rac1 with IQGAP1[49], much like activation of the carboxyl-terminal domain of CXCR2 stimulates the binding of activated Cdc42 to IQGAP1 to stimulate CXCR2 mediated chemotaxis. siRNA to IQGAP1 inhibits VEGF mediated cell motility. Moreover, Bensenor et al, demonstrated that FGF2 binding to FGFR1 stimulates IQGAP1-dependent lamellipodial protrusion and cell migration [48]. This event was coordinated by Arp2/3 and N-WASP which are also recruited to the lamellopodia. They showed that IQGAP1 binds to the cytoplasmic tail of FGFR1 and to N-WASP to stimulate the nucleation of branched actin filaments. We show here that the EGFR mediated effects on chemotaxis are also disrupted when the competing peptide fragment of IQGAP1 1-160 is expressed in cells. We also show for the first time the direct binding of IQGAP1 to the G protein-coupled receptor, CXCR2, and we show that knock-downof IQGAP1 inhibits CXCR2 mediated chemotaxis. Moreover, we clearly define for the first time the region of IQGAP1 that is involved in receptor binding to mediate chemotaxis and demonstrate that CXCR2 activation increases the association of Cdc42 to IQGAP1.

Finally, our characterization herein of CXCR2 interactions with several other proteins in the chemosynapse will greatly impact our current understanding of how the chemotactic response is relayed from activated chemokine receptors to the cytoskeleton and intracellular signaling cascades.

## Supporting Information

Figure S1 **No inhibition of IQGAP1-CXCR2 association when IQGAP1 1-160 fragment expressing in HEK-293 CXCR2 cells.** HEK293-CXCR2 cells were transiently transfected with either vector control or IQGAP1 1-160 expression construct. Cells were stimulated with either CXCL8 (100 ng/ml) or carrier buffer for 5 min at 37°C and co-immunoprecipitation was performed with anti-CXCR2 antibody conjugated to sepharose beads and immunoblotted with anti-IQGAP1 or CXCR2 antibody. Experiments were performed three times with similar results.
(TIF)

Figure S2 **No inhibition of IQGAP1-CXCR2 association in the presence of large amount of IQGAP1 1-160 fragment added in vitro in dHL-60 cells.** Differentiated HL-60 cells were lysed in lysis buffer (0.1% triton X-100 in PBS). Co-immunoprecipitation with anti-CXCR2 antibody was performed in the presence of a large amount of IQGAP1 fragment 1-160 amino acids (20 or 40 mg) or the non-competitive control fragment IQGAP1 161-431 or buffer only. IQGAP1 and CXCR2 were detected by immunoblot with anti-IQGAP1 or CXCR2, respectively. Experiments were repeated at least three times with similar results.
(TIF)

Figure S3 **No inhibition of IQGAP1-Cdc42 association when IQGAP1 1-160 fragment expressing in HEK-293 CXCR2 cells.** HEK293-CXCR2 cells were transiently transfected with either vector control or IQGAP1 1-160 expression construct. Cells were stimulated with either CXCL8 (100 ng/ml) or carrier buffer for 5 min at 37°C and co-immunoprecipitation was performed with anti-IQGAP1 polyclonal rabbit antibody (Santa Cruz, CA) and protein A/G agarose beads. Western blots were performed with anti-IQGAP1 or Cdc42 antibody, respectively. Experiments were repeated twice with similar results.
(TIF)

Figure S4 **Only N-terminal of IQGAP1 1-160 fragment associates with CXCR2.** The lysates of differentiated HL-60 cells were incubated with GST alone or various GST tagged fragments of IQGAP1. The GST pull-down products were resolved in a SDS-PAGE and detected by Western blot with anti-CXCR2 antibody (Santa Cruz Biotech, CA). Cell lysates were loaded as positive control and a ladder of fragments were observed due to the different forms of glycosylation of CXCR2 in differentiated HL-60 cells. CXCR2 was pulled down only with the GST tagged N-terminal and the GST-1-265 amino acid fragment of IQGAP1.
(TIF)
