# Supplementary figures and images for: IQGAP1 Is a Novel CXCR2-Interacting Protein and Essential Component of the “Chemosynapse”
Source: PLoS One. 2011 Aug 18;6(8):e23813. doi: 10.1371/journal.pone.0023813 (PMC3158102; doi:10.1371/journal.pone.0023813)

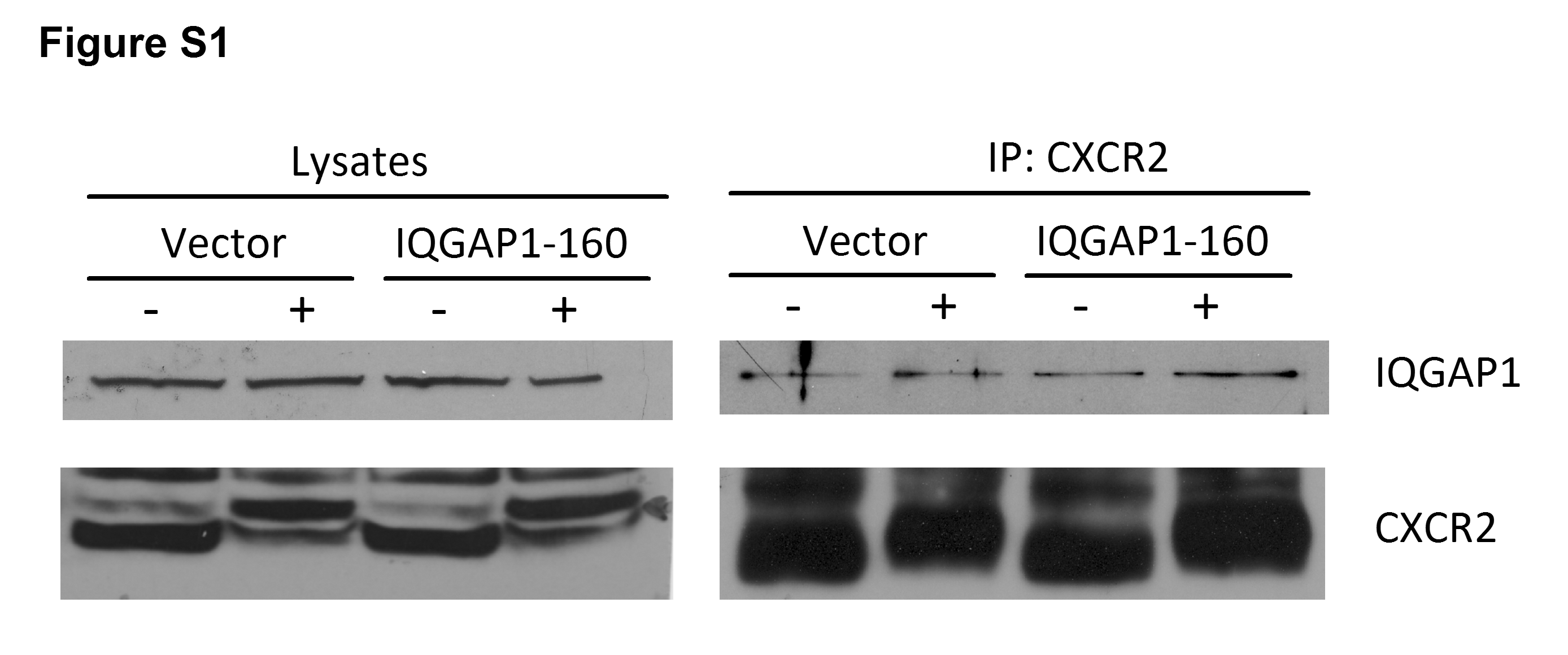

Supplement: Figure S1 — No inhibition of IQGAP1-CXCR2 association when IQGAP1 1-160 fragment expressing in HEK-293 CXCR2 cells. HEK293-CXCR2 cells were transiently transfected with either vector control or IQGAP1 1-160 expression construct. Cells were stimulated with either CXCL8 (100 ng/ml) or carrier buffer for 5 min at 37°C and co-immunoprecipitation was performed with anti-CXCR2 antibody conjugated to sepharose beads and immunoblotted with anti-IQGAP1 or CXCR2 antibody. Experiments were performed three times with similar results. (TIF) [file pone.0023813.s001.tif]

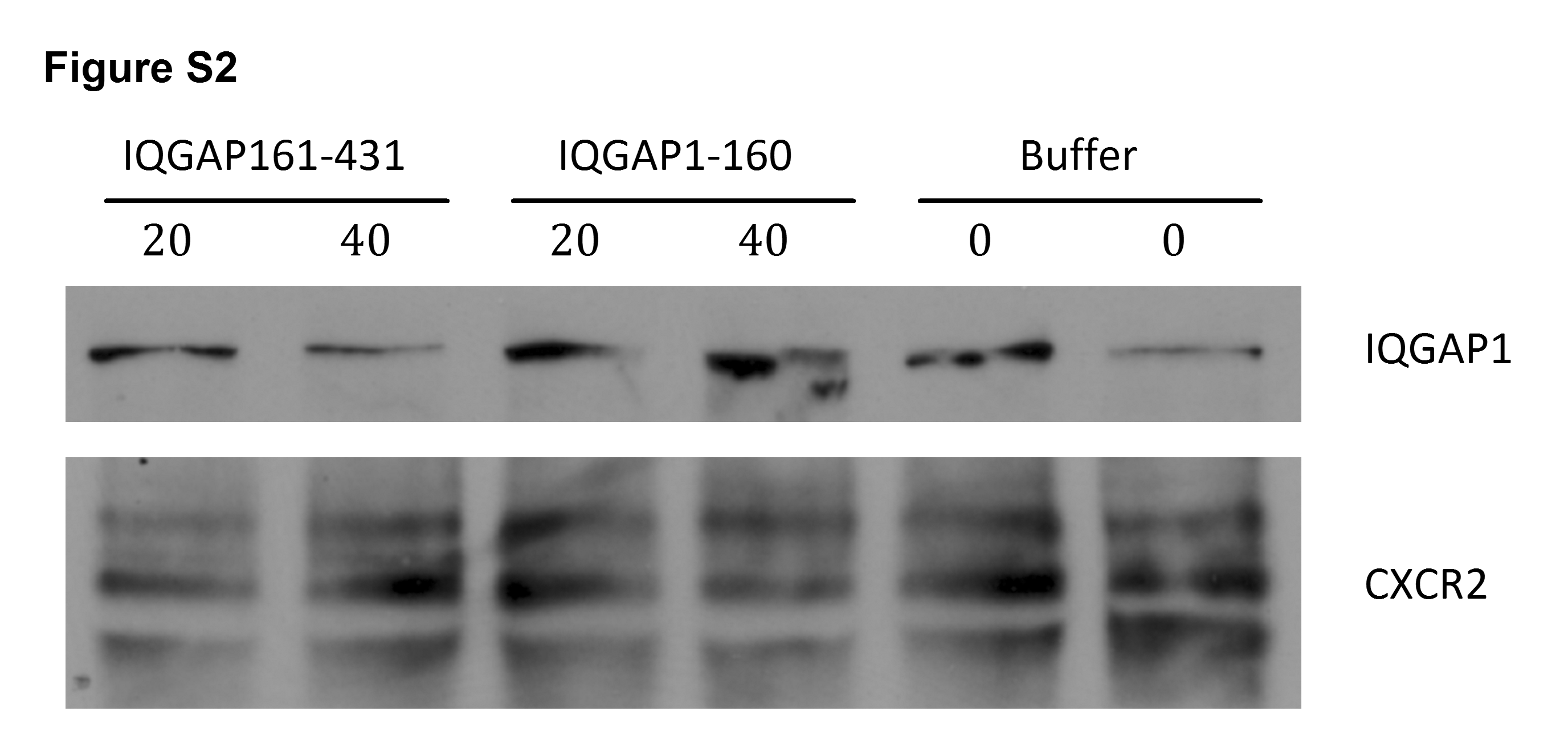

Supplement: Figure S2 — No inhibition of IQGAP1-CXCR2 association in the presence of large amount of IQGAP1 1-160 fragment added in vitro in dHL-60 cells. Differentiated HL-60 cells were lysed in lysis buffer (0.1% triton X-100 in PBS). Co-immunoprecipitation with anti-CXCR2 antibody was performed in the presence of a large amount of IQGAP1 fragment 1-160 amino acids (20 or 40 mg) or the non-competitive control fragment IQGAP1 161-431 or buffer only. IQGAP1 and CXCR2 were detected by immunoblot with anti-IQGAP1 or CXCR2, respectively. Experiments were repeated at least three times with similar results. (TIF) [file pone.0023813.s002.tif]

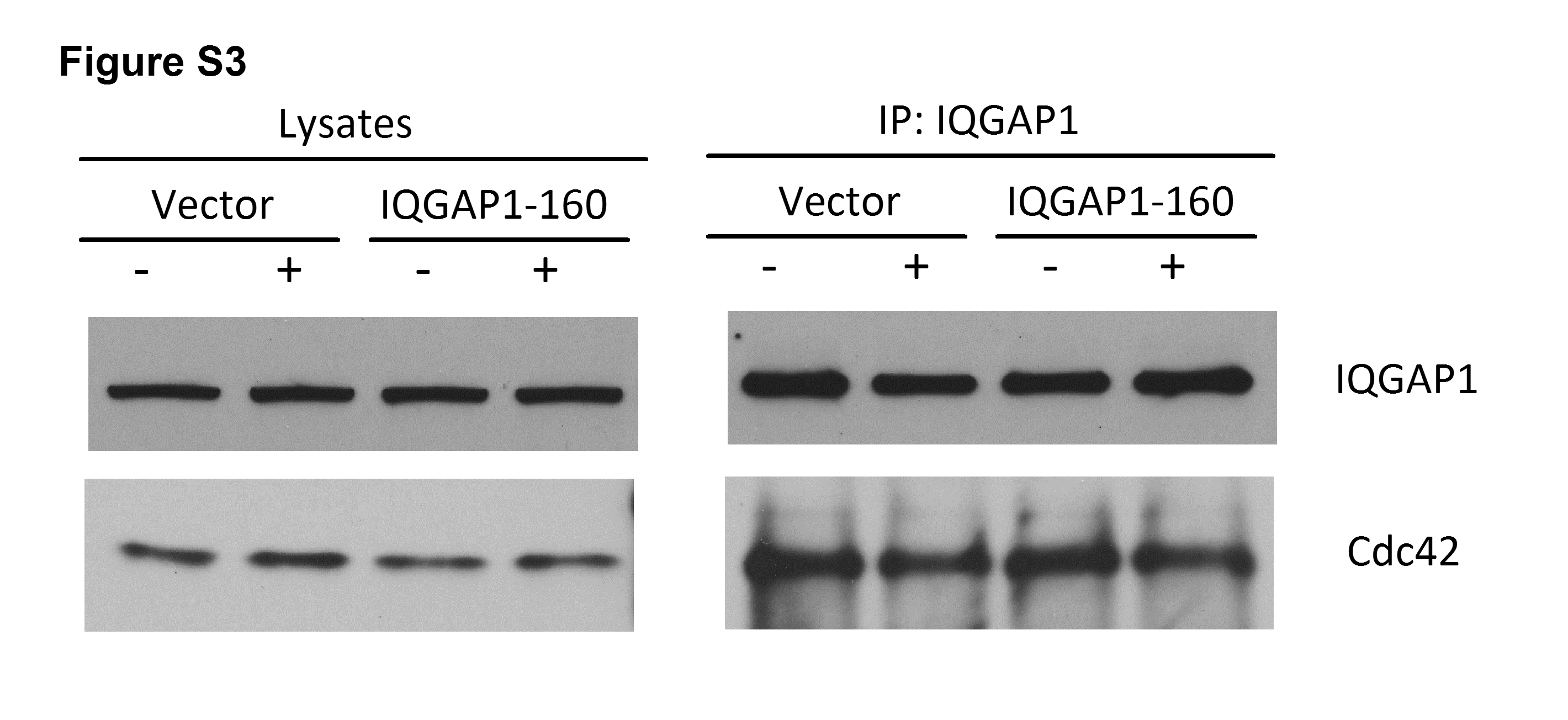

Supplement: Figure S3 — No inhibition of IQGAP1-Cdc42 association when IQGAP1 1-160 fragment expressing in HEK-293 CXCR2 cells. HEK293-CXCR2 cells were transiently transfected with either vector control or IQGAP1 1-160 expression construct. Cells were stimulated with either CXCL8 (100 ng/ml) or carrier buffer for 5 min at 37°C and co-immunoprecipitation was performed with anti-IQGAP1 polyclonal rabbit antibody (Santa Cruz, CA) and protein A/G agarose beads. Western blots were performed with anti-IQGAP1 or Cdc42 antibody, respectively. Experiments were repeated twice with similar results. (TIF) [file pone.0023813.s003.tif]

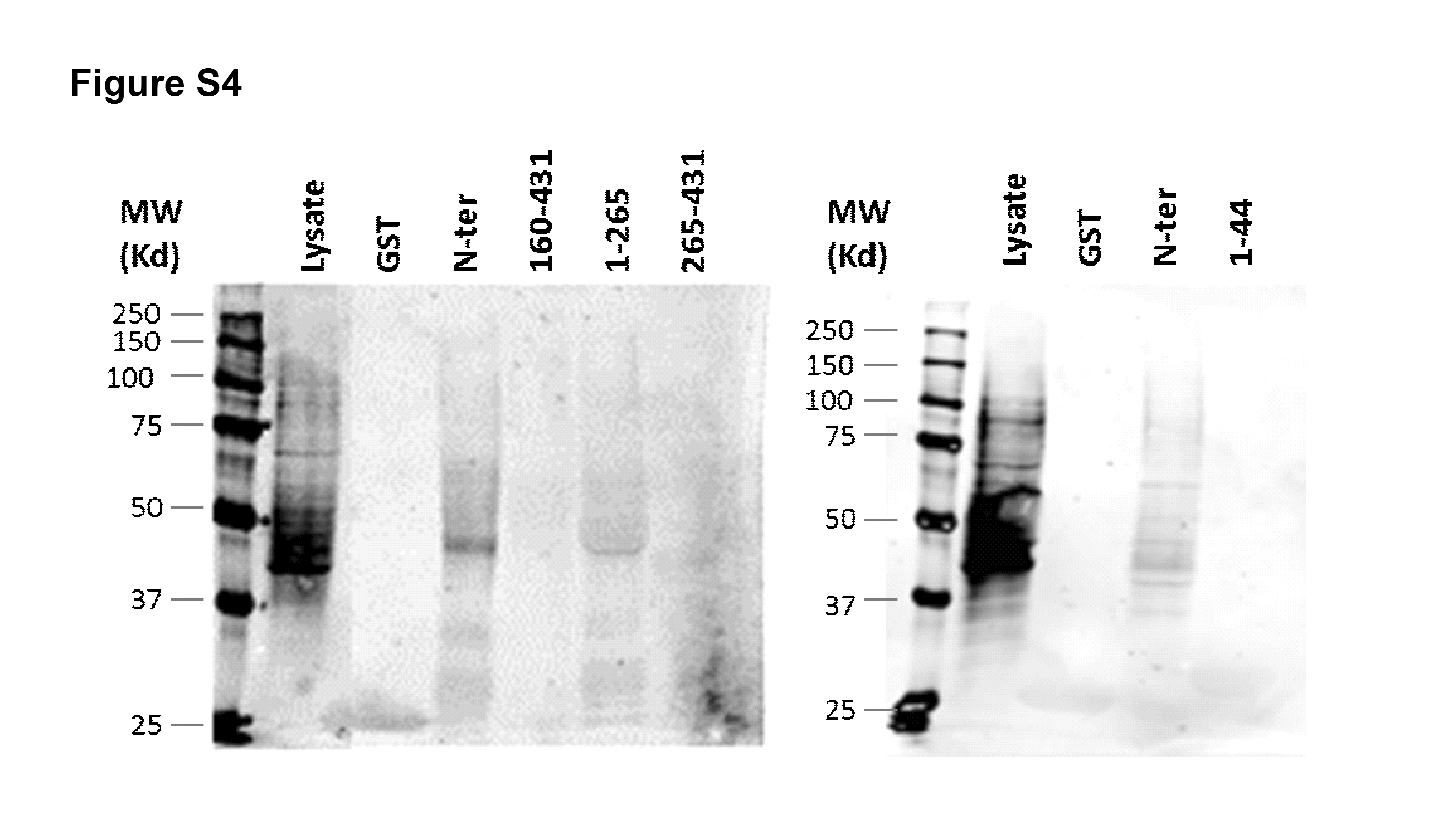

Supplement: Figure S4 — Only N-terminal of IQGAP1 1-160 fragment associates with CXCR2. The lysates of differentiated HL-60 cells were incubated with GST alone or various GST tagged fragments of IQGAP1. The GST pull-down products were resolved in a SDS-PAGE and detected by Western blot with anti-CXCR2 antibody (Santa Cruz Biotech, CA). Cell lysates were loaded as positive control and a ladder of fragments were observed due to the different forms of glycosylation of CXCR2 in differentiated HL-60 cells. CXCR2 was pulled down only with the GST tagged N-terminal and the GST-1-265 amino acid fragment of IQGAP1. (TIF) [file pone.0023813.s004.tif]
